# The Effect of Adenosine A_2A_ Receptor Antagonists on Hydroxyl Radical, Dopamine, and Glutamate in the Striatum of Rats with Altered Function of VMAT2

**DOI:** 10.1007/s12640-012-9316-9

**Published:** 2012-03-10

**Authors:** Krystyna Gołembiowska, Anna Dziubina

**Affiliations:** Institute of Pharmacology, Polish Academy of Sciences, 31-343 Kraków, 12 Smętna Street, Kraków, Poland

**Keywords:** Parkinson’s disease, Adenosine A_2A_ receptor antagonists, VMAT2 function, Oxidative stress

## Abstract

It has been shown that a decreased vesicular monoamine transporter (VMAT2) function and the disruption of dopamine (DA) storage is an early contributor to oxidative damage of dopamine neurons in Parkinson’s disease (PD). In our previous study, we demonstrated that adenosine A_2A_ receptor antagonists suppressed oxidative stress in 6-hydroxydopamine-treated rats suggesting that this effect may account for neuroprotective properties of drugs. In the present study, rats were injected with reserpine (10 mg/kg sc) and 18 h later the effect of the adenosine A_2A_ receptor antagonists 8-(3-chlorostyryl)caffeine (CSC) and 4-(2-[7-amino-2-(2-furyl)[1,2,4]triazolo[2,3-*a*][1,3,5]triazin-5-ylamino]ethyl)phenol (ZM 241385) on extracellular DA, glutamate and hydroxyl radical formation was studied in the rat striatum using in vivo microdialysis. By disrupting VMAT2 function, reserpine depleted DA stores, and increased glutamate and hydroxyl radical levels in the rat striatum. CSC (1 mg/kg) but not ZM 241385 (3 mg/kg) increased extracellular DA level and production of hydroxyl radical in reserpinised rats. Both antagonists decreased the reserpine-induced increase in extracellular glutamate. l-3,4-Dihydroxyphenylalanine (L-DOPA) (25 mg/kg) significantly enhanced extracellular DA, had no effect on reserpine-induced hydroxyl radical production and decreased extracellular glutamate concentration. CSC but not ZM 241385 given jointly with L-DOPA increased the effect of L-DOPA on extracellular DA and augmented the reserpine-induced hydroxyl radical production. CSC and ZM 241385 did not influence extracellular glutamate level, which was decreased by L-DOPA. It seems that by decreasing the MAO-dependent DA metabolism rate, CSC raised cytosolic DA and by DA autoxidation, it induced hydroxyl radical overproduction. Thus, the methylxanthine A_2A_ receptor antagonists bearing properties of MAO-B inhibitor, like CSC, may cause a risk of oxidative stress resulting from dysfunctional DA storage mechanism in early PD.

## Introduction

Progressive degeneration of the dopamine-containing neurons in the substantia nigra pars compacta results in deficiency of striatal dopamine (DA) and loss of neurochemical transport systems, such as the dopamine transporter (DAT) and the vesicular monoamine transporter (VMAT2) (Miller et al. 1997, 1999). PET studies in a non-human primate model of Parkinson’s disease (PD) showed that a decreased VMAT2 function and the disruption of DA sequestration was an early and potent contributor to oxidative damage of dopamine neurons in PD pathogenesis (Chen et al. 2008).

DA is a highly reactive molecule that is capable of autoxidation to a quinone in the basic pH of the cytosol. Furthermore, cytosolic MAO-dependent DA metabolism leads to the formation of aldehydes and peroxides (Halliwell 2006). Regarding the role of oxidative stress in the pathogenesis of PD, packing of cytosolic DA into synaptic vesicles by VMAT2 prevents its autoxidation and subsequent degeneration of dopamine neurons. An animal model of PD mimicking the altered DA homeostasis by impaired DA storage mechanisms is based on the administration of the irreversible VMAT2 inhibitor reserpine to rats (Carlsson et al. 1957). Reserpine reduces vesicular storage and release of brain monoamines and leads to the accumulation of oxidative products of neurotransmitters (Caudle et al. 2007).

Recently, antagonists of adenosine A_2A_ receptors appeared as a new promising non-dopaminergic therapy of PD. Striatopallidal neurons are highly enriched in adenosine A_2A_ receptors which occur there as heteromeric complexes with dopamine D_2_ receptors (Ferré et al. 1993). Behavioral studies in rodents and in non-human primates showed that A_2A_ receptor antagonists reversed motor impairment induced by 6-hydroxydopamine (6-OHDA) or MPTP (Schwarzschild et al. 2006; Morelli et al. 2007). The mechanism of antiparkinsonian effects of A_2A_ receptor antagonists is based on their ability to modulate GABA release and DA-dependent *c*-*fos* activation in the indirect striatopallidal pathway (Pollack and Fink 1995; Ochi et al. 2000). In addition, presynaptic A_2A_ receptors are able to control corticostriatal glutamatergic transmission by counteracting D_2_ receptor function (Tozzi et al. 2007). Several epidemiological and animal studies have suggested neuroprotective effects of caffeine and selective A_2A_ adenosine receptor antagonists (Ross et al. 2000; Ascherio et al. 2001; Xu et al. 2005; Chen et al. 2007). A protective effect of caffeine and more selective antagonists of A_2A_ receptors, similar to genetic inactivation of A_2A_ receptors, was observed in an animal MPTP neurotoxicity model (Xu et al. 2005; Chen et al. 2007) or in ischemia and excitotoxic brain injury models (Popoli et al. 2004; Chen et al. 2007). The mechanism allowing A_2A_ receptor antagonists to protect dopaminergic neurons has not been fully explained yet, but a variety of their effects on various types of neurons, e.g., glutamatergic nerve terminals and glial or immune cells, suggest its complex nature (Chen et al. 2007). In our earlier study, we have shown that A_2A_ receptor antagonists decreased the production of free radical and lowered extracellular glutamate level in 6-OHDA-treated rats (Gołembiowska et al. 2009; Gołembiowska and Dziubina 2012). Moreover, A_2A_ receptor antagonists administered in combination with l-3,4-Dihydroxyphenylalanine (L-DOPA) did not change inhibitory effect of L-DOPA on free radical generation and glutamate enhancement in the striatum of 6-OHDA-treated rats (Gołembiowska and Dziubina 2012).

A class of A_2A_ antagonists belonging to methylxanthine derivatives offers a neuroprotective benefit as MAO-B inhibitors (Castagnoli et al. 2003). Inhibition of DA degradation by MAO-B attenuates hydrogen peroxide formation, but at the same time it increases the risk of DA autoxidation resulting from augmentation of the cytosolic DA pool. Therefore, in our present study, we aimed to investigate whether two A_2A_ receptor antagonists 8-(3-chlorostyryl)caffeine (CSC) and 4-(2-[7-amino-2-(2-furyl)[1,2,4]triazolo[2,3-*a*][1,3,5]triazin-5-ylamino]ethyl)phenol (ZM 241385) belonging to different chemical classes (methylxanthine and non-xanthine derivatives, respectively) can modulate extracellular level of DA and glutamate as cellular sources of hydroxyl radical in animals with reduced VMAT2 function after reserpine administration. Since L-DOPA may be toxic in the brain by promoting the formation of reactive species and neurotoxic quinones when cytosolic level of DA increases after disruption of DA storage mechanisms (Halliwell 2006), we also studied the effect of A_2A_ receptor antagonists given in combination with L-DOPA in rats treated with reserpine.

## Materials and Methods

### Animals

Microdialysis studies were conducted in male Wistar rats (250–300 g), bred at the Institute of Pharmacology, Polish Academy of Sciences, Krakow, Poland. The rats were housed in temperature- and humidity-controlled rooms on a 12-h light/dark cycle, with free access to filtered tap water and standard pelleted laboratory chow throughout the study. The experimental procedures and housing conditions used were in strict accordance with the Polish legal regulations concerning experiments on animals (Dz. U. 05.33.289). All the experimental protocols were approved by the Local Bioethics Commission for Animal Experiments.

### Drugs

L-DOPA, CSC, benserazide, and *p*-hydroxybenzoic acid (PBA) were obtained from Sigma-Aldrich (Poznań, Poland), reserpine was obtained from Fluka-Analytical, Poland), whereas 4-(2-[7-amino-2-(2-furyl)[1,2,4]triazolo[2,3-*a*][1,3,5]triazin-5-ylamino]ethyl)phenol (ZM 241385) came from TOCRIS (Warsaw, Poland). All the chemicals used for HPLC were purchased from Merck (Warsaw, Poland). L-DOPA and benserazide were dissolved in saline. A solution of PBA was prepared in an artificial cerebrospinal fluid (aCSF) and was then adjusted to pH 7.4 with 0.1 M NaOH. CSC was initially dissolved in dimethyl sulfoxide (DMSO; Sigma-Aldrich, Poznań, Poland) and was then diluted in at least 20 vols. of the vehicle consisting of a 20:80 (v/v) mixture of Alkamulus EL-620 (Rhone-Poulenc, Cranbury, NJ) and a phosphate-buffered saline. ZM 241385 was dissolved in a small amount of DMSO and then was diluted in Cremophor EL (Sigma-Aldrich, Poznań, Poland) and 0.9% NaCl (final concentration: a 15% DMSO and a 15% Cremophor EL). All injections were made by an intraperitoneal route (i.p.). Reserpine dissolved in a mixture of benzyl alcohol (2 ml), citric acid (250 mg), and Tween-80 in H_2_O was given in a dose of 10 mg/kg 18 h before microdialysis experiment. CSC (1 mg/kg) and ZM 241385 (3 mg/kg) were given in single injections as indicated in the figures. L-DOPA (25 mg/kg) was injected 20 min after the administration of A_2A_ receptor antagonists together with benserazide (12.5 mg/kg). Control animals received respective vehicles.

### Determination of Monoamine Levels

For the measurement of dopamine (DA), 3,4-dihydroxyphenylacetic acid (DOPAC) and homovanilic acid (HVA), the brain tissue was homogenized in 0.1 M HClO_4_, centrifuged at 4°C for 5 min at 10,000×*g*, and the supernatant was filtered through 0.1 μm (Millipore) membranes. An aliquot of 2–5 μl of each sample was then injected into a high performance liquid chromatograph (HPLC) with electrochemical detection.

### In Vivo Microdialysis

The rats were anaesthetized with ketamine (75 mg/kg i.m.) and xylazine (10 mg/kg i.m.) and placed in a stereotaxic apparatus (David Kopf Instruments, Tujunga, CA, USA). Their skulls were exposed and small holes were drilled for the insertion of microdialysis probes into the striatum using the following coordinates: 1.8 mm anterior from the bregma; 2.8 mm lateral from the sagittal suture; −7.0 mm ventral from the dura (Paxinos and Watson 1998). Vertical microdialysis probes were constructed as described in detail elsewhere (Gołembiowska et al. 2009). Probe inlets were connected to a syringe pump (BAS, IN, USA) which delivered an aCSF composed of [mM]: NaCl 147, KCl 4.0, MgCl_2_ 1.0, CaCl_2_ 2.2; pH 7.4 at a flow rate of 2 μl/min. All metal parts of the aCSF delivery system were replaced with PEEK components or were passivated with 6 M HNO_3_. Baseline samples were collected every 20 min after the washout period to obtain a stable extracellular neurotransmitter level. Appropriate drugs were then administered 20 min before L-DOPA injection given at time 0, as shown in figures, and dialysate fractions were collected for 240 min. At the end of the experiment, the rats were killed and their brains were histologically examined to validate probe placement.

### Analytical Procedure

DA, DOPAC, and HVA were analyzed by HPLC with an electrochemical detection. The level of hydroxyl radicals was estimated as 3,4-dihydroxybenzoic acid (3,4-DHBA), a product of the spin trap reagent PBA (1 mM) applied via the microdialysis probe. DA and its metabolites were simultaneously determined in the same fractions of striatal dialysates. Chromatography was performed using an LC-10 AD pump (Shimadzu Europa GmbH, Warsaw, Poland), an LC-4B amperometric detector with a cross-flow detector cell (BAS, IN, USA) and a BDS-Hypersil C18 analytical column (3 × 100 mm, 3 μm; Thermo Electron Corp., UK). The mobile phase consisted of 0.1 M monochloroacetic acid adjusted to pH 3.7 with 3 M sodium hydroxide, 0.5 mM EDTA, 13 mg/l 1-octanesulfonic acid sodium salt, a 5.7% methanol, and a 0.8% acetonitrile. The flow rate was 0.5 ml/min, and the applied potential of a 3-mm glassy carbon electrode was +600 mV at a sensitivity of 2 nA/V. Concentrations of all compounds were calculated by comparing their peak areas with respective standards and were processed by Chromax 2001 (Pol-Lab, Warsaw, Poland) software run on a personal computer. The obtained values were not corrected for in vitro probe recovery, which was approximately 10–15%.

Glutamate was measured in dialysates (20 μl) after derivatization with 4-dimethylaminoazobenzene-4′-sulfonylchloride (DABS-Cl) at 70°C for 12 min, according to Knecht and Chang (1986). Dabsylated amino acids were separated on an Ultrasphere ODS (4.6 × 150 mm, 3 μm) column (Supelco, Poznań, Poland) by gradient elution, with solvent A (10 mM citric acid, 4% dimethylformamide) and solvent B (acetonitrile). Dabsylated compounds were detected by measuring an absorbance at 436 nm using Beckman Amino Acid System Gold with VIS detection.

### Data Analysis

All obtained data are given in absolute numbers. The statistical significance of differences between experimental groups was calculated using a one-way ANOVA for repeated-measures, followed by Tukey’s post hoc test. The results were considered statistically significant at *P* < 0.05.

## Results

### The Effects of Reserpine on DA, DOPAC, HVA in the Rat Striatum

Reserpine produced a substantial depletion of DA and changed the level of its metabolites DOPAC and HVA 24 h after the injection (Table 1). Dialysate level of DA was decreased by ca. 96%, while DOPAC and HVA extracellular concentrations were increased by 44 and 11% of control, respectively. The content of striatal DA was attenuated by ca. 95%, while DOPAC and HVA contents were increased by 33 and 58%, respectively. These results indicate reserpine-induced damage of intracellular DA stores and increase in DA turnover.

**Table 1:** Tissue content and dialysate level of DA, DOPAC, and HVA in the striatum of control and reserpine (10 mg/kg)-treated rats

	Mean ± SEM (*n*)		
	DA (pg/10 μl)	DOPAC (ng/10 μl)	HVA (ng/10 μl)
Dialysate			
Control	10.5 ± 0.53 (43)	2.8 ± 0.11 (39)	1.9 ± 0.05 (39)
Reserpine	0.4 ± 0.06 (44)*	4.0 ± 0.07 (36)*	2.11 ± 0.06 (39)*
% of control	3.9	144	111

	DA (ng/mg wt)	DOPAC (ng/mg wt)	HVA (ng/mg wt)
Tissue			
Control	12,442 ± 956 (12)	1,331 ± 76 (12)	870 ± 118 (12)
Reserpine	568 ± 99 (12)*	1,773 ± 327 (12)*	1,378 ± 281 (12)*
% of control	5	133	158

* *P* < 0.01 versus control

### The Effects of CSC and ZM 241385 on Extracellular Level of DA, Glutamate and Production of Hydroxyl Radical in Reserpine-Treated Rats

**Fig. 1:**
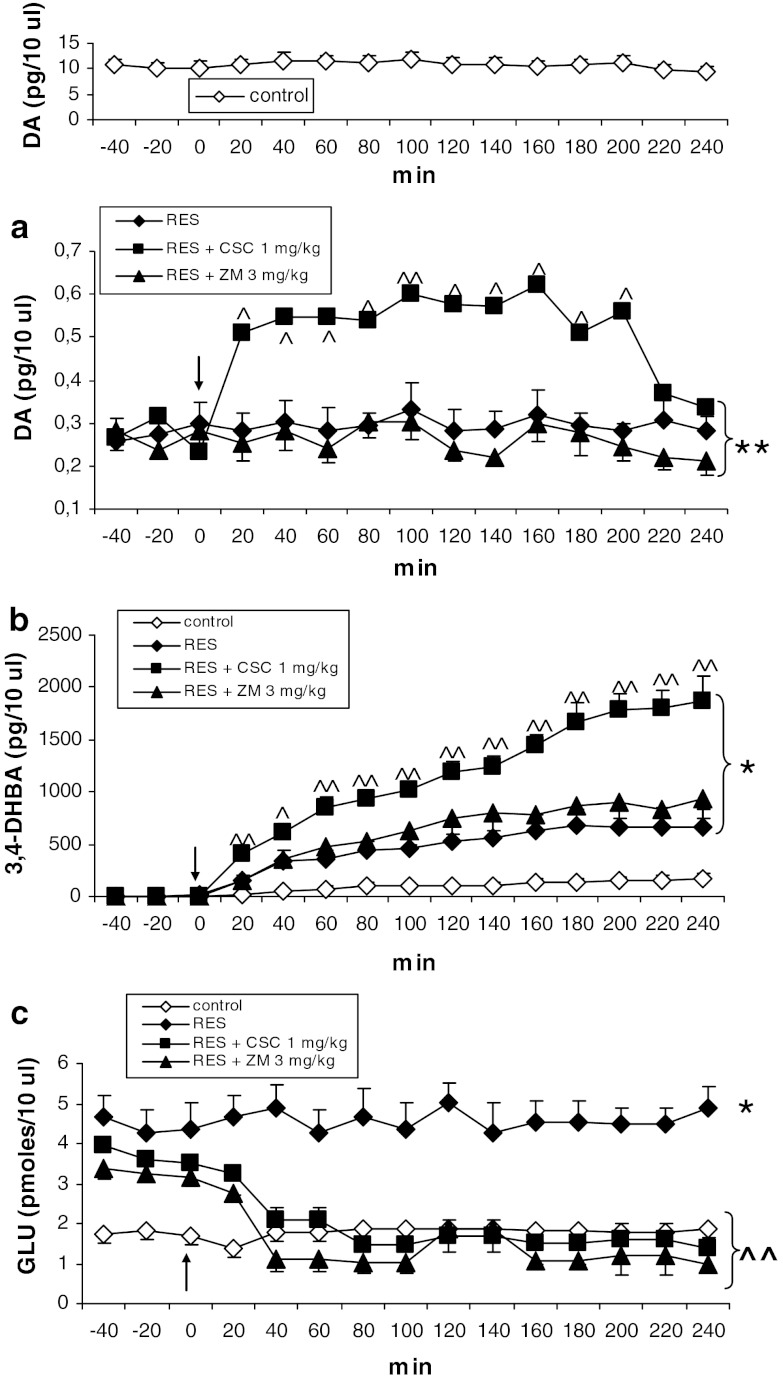
The effects of CSC (1 mg/kg) and ZM 241385 (3 mg/kg) on extracellular concentrations of DA (**a**), hydroxyl radical (3,4-DHBA, **b**) and glutamate (GLU, **c**) in the striatum of rats treated with reserpine (10 mg/kg). The injection of drugs is indicated by an *arrow*. The data are the mean ± SEM (*n* = 5–7). *P < 0.05; **P < 0.01 versus control; ^^^ *P* < 0.05; ^^^^ *P* < 0.01 versus reserpine

CSC (1 mg/kg) increased, while ZM 241385 (3 mg/kg) did not influence extracellular level of DA in the rat striatum attenuated by reserpine (Fig. 1a). Repeated- measures ANOVA showed a significant effect of treatment (*F*_3,15_ = 37.63, *P* = 0), but a non-significant effect of time (*F*_14,210_ = 1.53, *P* = 0.103), and no interaction between both factors (*F*_42,210_ = 1.099, *P* = 0.33). Post hoc analysis with Tukey’s test showed that CSC significantly increased extracellular DA level from 20 to 200 min (*P* < 0.05–0.01 in comparison with reserpine).

Hydroxyl radical production was increased in the striatum of reserpinized rats (Fig. 1b). CSC (1 mg/kg), but not ZM 241385 (3 mg/kg), further increased its production (Fig. 1b). Repeated-measures ANOVA showed a significant effect of treatment (*F*_3,18_ = 24.21, *P* = 0.00001), time (*F*_11,198_ = 22.56, *P* = 0), and an interaction between both factors (*F*_33,198_ = 5.27, *P* = 0). Post hoc analysis with Tukey’s test showed that CSC significantly increased production of hydroxyl radical from 20 to 240 min after the administration (*P* < 0.05–0.01 in comparison with reserpine).

Extracellular glutamate level was increased by reserpine in comparison with control group (Fig. 1c). CSC (1 mg/kg) and ZM 241385 (3 mg/kg) decreased the reserpine-enhanced extracellular glutamate level, to control values (Fig. 1c). Repeated-measures ANOVA showed a significant effect of treatment (*F*_3,12_ = 6.79, *P* = 0.006), time (*F*_14,168_ = 4.46, *P* = 0.00001) and an interaction between both factors (*F*_42,168_ = 1.97, *P* = 0.001). Post hoc analysis with Tukey’s test showed that CSC and ZM 241385 significantly decreased extracellular glutamate level from 40 to 240 min after administration (*P* < 0.01 in comparison with reserpine).

#### The Effect of L-DOPA on Extracellular Level of DA, DOPAC, HVA, Glutamate and Production of Hydroxyl Radical in Reserpine-Treated Rats

**Fig. 2:**
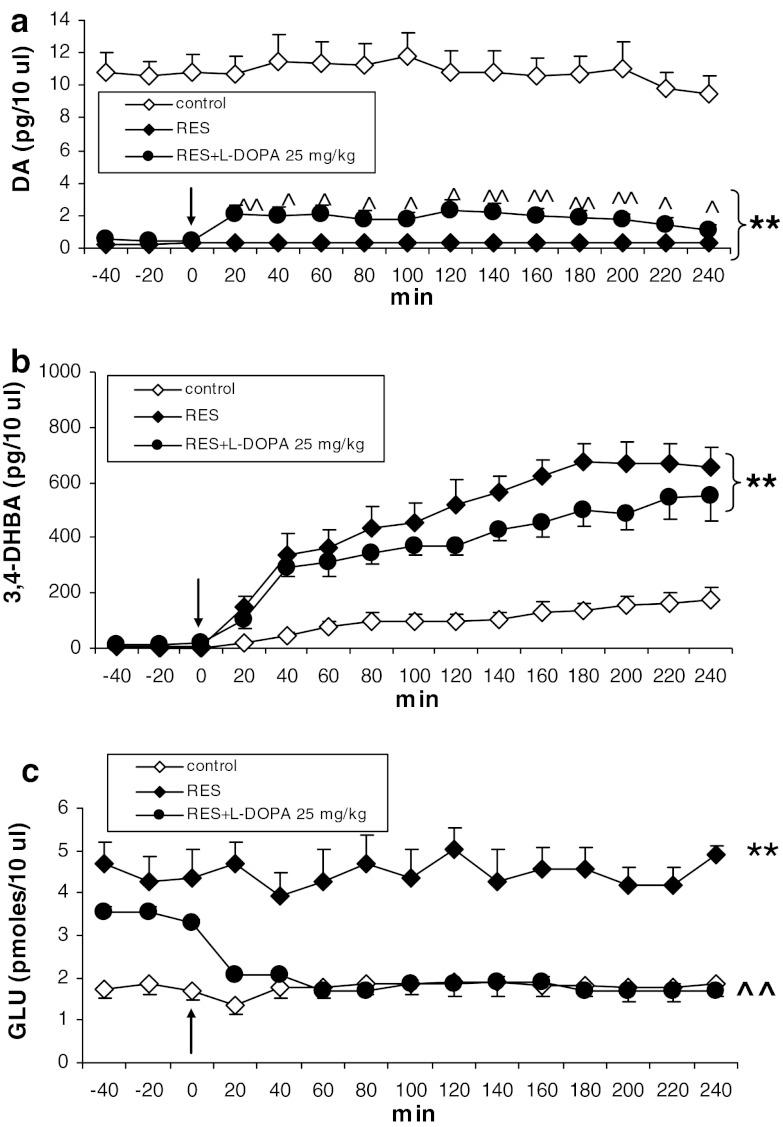
The effect of L-DOPA (25 mg/kg) on extracellular concentrations of DA (**a**), hydroxyl radical (3,4-DHBA, **b**) and glutamate (GLU, **c**) in the striatum of rats treated with reserpine (10 mg/kg). The injection of L-DOPA is indicated by an *arrow*. The data are the mean ± SEM (*n* = 5–7). ** < 0.0*P*1 versus control; ^^^ *P* < 0.05; ^^^^ *P* < 0.01 versus reserpine

**Fig. 3:**
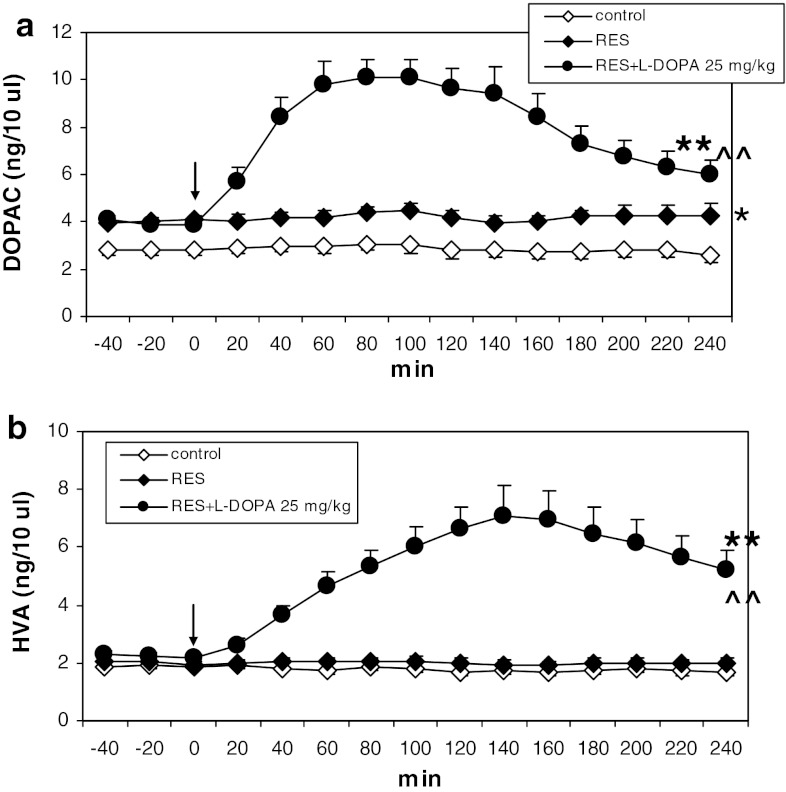
The effect of L-DOPA (25 mg/kg) on extracellular concentrations of DOPAC (**a**), and HVA (**b**) in the striatum of rats treated with reserpine (10 mg/kg). The injection of L-DOPA is indicated by an *arrow*. The data are the mean ± SEM (*n* = 5–7). *P < 0.05; **P < 0.01 versus control; ^^^^ *P* < 0.01 versus reserpine

L-DOPA (25 mg/kg) increased the level of DA, DOPAC, and HVA in the striatum of reserpinized rats (Figs. 2a, 3a, b). Repeated-measures ANOVA showed a significant effect of treatment on DA (*F*_2,11_ = 30.55, *P* = 0.00003), time (*F*_14,154_ = 2.34, *P* = 0.006), but no interaction for both factors (*F*_28,154_ = 1.49, *P* = 0.07). Post hoc analysis with Tukey’s test showed that L-DOPA significantly increased extracellular DA concentration (*P* < 0.05–0.01) in comparison with reserpine. Repeated- measures ANOVA showed a significant effect of treatment on DOPAC (*F*_2,14_ = 20.66, *P* = 0.0001), time (*F*_14,196_ = 19.75, *P* = 0) and interaction for both factors (*F*_28,196_ = 18.85, *P* = 0). Post hoc analysis with Tukey’s test showed that L-DOPA significantly increased extracellular DOPAC (*P* < 0.01) in comparison with reserpine. Repeated-measures ANOVA showed a significant effect of treatment on HVA (*F*_2,14_ = 18.71, *P* = 0.0001), time (*F*_14,196_ = 12.9, *P* = 0), and interaction for both factors (*F*_28,196_ = 18.43, *P* = 0). Post hoc analysis with Tukey’s test showed that L-DOPA significantly increased extracellular HVA (*P* < 0.01) in comparison with reserpine.

Hydroxyl radical production, increased by reserpine, was not changed in rats treated with L-DOPA (Fig. 2b). Repeated-measures ANOVA showed a significant effect of treatment (*F*_2,13_ = 4.47, *P* = 0.03), time (*F*_11,143_ = 19.55, *P* = 0) and an interaction between both factors (*F*_22,143_ = 1.71, *P* = 0.03). Post hoc analysis with Tukey’s test showed a significant difference (*P* < 0.01) between control and reserpine or control and a combination of reserpine and L-DOPA (Fig. 2b).

Extracellular glutamate concentration, increased by reserpine, was decreased by L-DOPA to the control level (Fig. 2c). Repeated-measures ANOVA showed a significant effect of treatment (*F*_2,10_ = 7.52, *P* = 0.01), there was no significant effect of time (*F*_14,140_ = 1.27, *P* = 0.23) and there was an interaction between both factors (*F*_28,140_ = 1.73, *P* = 0.02). Post hoc analysis with Tukey’s test showed a significant effect of L-DOPA on extracellular glutamate level (*P* < 0.01).

#### The Effects of CSC and ZM 241385 Administered Together with L-DOPA on Extracellular Level of DA, Glutamate and Production of Hydroxyl Radical in Reserpine-Treated Rats

**Fig. 4:**
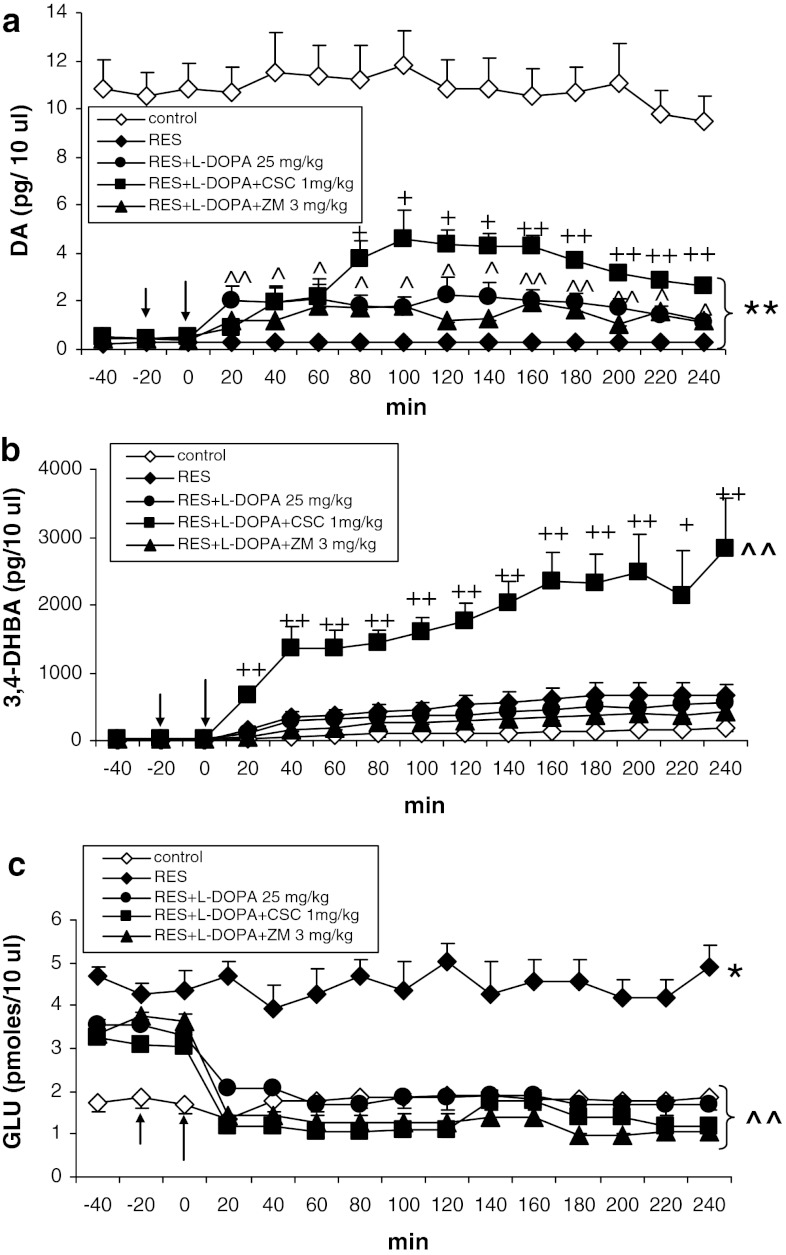
The effects of CSC (1 mg/kg) and ZM 241385 (3 mg/kg) on L-DOPA (25 mg/kg)-induced change in extracellular level of DA (**a**), hydroxyl radical (3,4-DHBA, **b**), and glutamate (GLU, **c**) in the striatum of rats treated with reserpine (10 mg/kg). The injection of drugs is indicated by an *arrow*. The data are the mean ± SEM (*n* = 5–7). *P < 0.05; **P < 0.01 versus control; ^^^ *P* < 0.05; ^^^^ *P* < 0.01 versus reserpine; ^+^ *P* < 0.05; ^++^ *P* < 0.01 versus reserpine + L-DOPA

CSC (1 mg/kg), but not ZM 241385 (3 mg/kg) enhanced extracellular DA level, increased by L-DOPA (25 mg/kg) (Fig. 4a). Repeated-measures ANOVA showed a significant effect of treatment (*F*_3,16_ = 4.04, *P* = 0.03), time (*F*_14,224_ = 7.73, *P* = 0), and an interaction between both factors (*F*_42,224_ = 2.89, *P* = 0). Post hoc analysis with Tukey’s test showed a significant effect of L-DOPA (*P* < 0.05–0.01) in comparison with the reserpine group and a significant effect of CSC (*P* < 0.05–0.01) in comparison with rats treated with reserpine and L-DOPA. The L-DOPA-increased extracellular levels of DOPAC and HVA were unchanged by CSC and ZM 241385 (results not shown).

Production of hydroxyl radical was markedly increased by CSC (1 mg/kg) given to rats treated with reserpine and L-DOPA, but there was no effect of ZM 241385 (Fig. 4b). Repeated-measures ANOVA showed a significant effect of treatment (*F*_4,22_ = 15.69, *P* = 0.00001), time (*F*_11,242_ = 11.34, *P* = 0), and there was an interaction between both factors (*F*_44,242_ = 2.86, *P* = 0). Post hoc analysis with Tukey’s test showed significant effect of CSC (*P* < 0.05–0.01).

Extracellular glutamate was decreased by L-DOPA, CSC (1 mg/kg), and ZM 241385 (3 mg/kg) to control level (Fig. 4c). Repeated-measures ANOVA showed a significant effect of treatment (*F*_4,20_ = 11.46, *P* = 0.00005), time (*F*_14,280_ = 15.38, *P* = 0), and interaction between both factors (*F*_56,280_ = 3.34, *P* = 0). Post hoc analysis with Tukey’s test showed a significant effect of CSC, ZM 241385, and L-DOPA (*P* < 0.01) in comparison with reserpine.

## Discussion

Numerous animal studies and clinical trials have demonstrated that A_2A_ receptor antagonists are able to improve motor deficits in advanced PD (Xu et al. 2005; Jenner et al. 2009). Later, experimental and epidemiologic evidence suggested neuroprotective potential for A_2A_ receptor antagonists (Chen et al. 2007; Jenner et al. 2009). Thus, these drugs used early in the therapy of PD might slow or halt degeneration of dopaminergic neurons. The neuroprotective mechanism of A_2A_ antagonists is not fully understood, but our former study showed that A_2A_ antagonists decreased free radical production and indicated that overactive glutamate neurotransmission might be the source of oxidative stress in the animal model of PD in which nigrostriatal neurons were damaged with 6-OHDA (Gołembiowska and Dziubina 2012). The data of the present study show that reserpine, by irreversibly blocking VMAT2, caused DA depletion in the rat striatum by ca. 95% in extraneuronal space as well as in intraneuronal DA pool. In contrast to the decrease in DA, the level of extraneuronal and intraneuronal DOPAC and HVA was increased, indicating on accelerated turnover in existing dopamine neurons. An increased MAO-catalyzed DA metabolism yields hydrogen peroxide for Fenton reaction while DA in slightly basic pH of the cytosol undergoes autoxidation into quinones (Asanuma et al. 2003). The inhibition of VMAT2 is similar to the beginning of PD as shown by Chen et al. (2008), who reported that MPTP administration into non-human primates induced a decrease in the striatal VMAT2 which preceded nigrostriatal dopamine neuron degeneration. Our data indicate that dysfunction of DA storage induced by reserpine promotes the formation of DA-oxidative products and leads to generation of free radicals. Furthermore, the decrease in synaptic DA and the lack of inhibitory control of presynaptic D_2_ receptors on cortico-striatal terminals (Donzanti et al. 1993) results in augmentation of glutamate release. Excitotoxicity due to overactive glutamate neurotransmission is able to accelerate extracellular free radical formation (Morari et al. 1998).Thus, dysfunction of DA storage and overactive glutamate neurotransmission caused by reserpine may induce an increase in free radical production. Both A_2A_ receptor antagonists, CSC and ZM 241385 used in our study in reserpine-treated rats by the blockade of adenosine A_2A_ receptor normalized glutamate neurotransmission in cortico-striatal terminals. However, CSC and ZM 241385 differed in their effect on the striatal DA, since CSC, but not ZM 241385 increased DA release in the striatum of reserpine-treated rats. As mentioned in the introduction, CSC is not only A_2A_ receptor antagonist, but also effectively inhibits MAO-B activity. This later CSC capacity may increase the cytosolic DA and by its autoxidation may lead to the increased hydroxyl radical formation, especially in rats with dysfunctional VMAT2. ZM 241385, devoid of the effect on MAO-B activity, did not influence DA concentration and hydroxyl radical formation. The restoration of glutamate neurotransmission by CSC and ZM 241385 does not seem to influence generation of hydroxyl radical in reserpine-treated rats—these results are different in comparison with our earlier study, where in the absence of dopamine innervation, the increased glutamate seemed to be responsible for free radical production (Gołembiowska and Dziubina 2012).

L-DOPA in a single dose of 25 mg/kg effectively increased extracellular DA level, inhibited reserpine-induced increase in extracellular glutamate, but did not influence hydroxyl radical formation. Normalization of glutamate release seems to be mediated via D_2_ receptor stimulated by L-DOPA-derived DA, as shown also by others (Meshul et al. 2000; Jonkers et al. 2002). However, the lack of L-DOPA effect on the increased hydroxyl radical production by reserpine indicates that glutamate does not seem to be an essential source of free radicals in this acute animal model of PD. It suggests that the accelerated DA metabolism in dopaminergic neurons observed in rats with dysfunctional VMAT2 and a marked increase of DOPAC and HVA extracellular concentration may account for the high level of hydroxyl radical in spite of L-DOPA normalized glutamate release. This is in opposition to our earlier study where an enhanced glutamate and hydroxyl radical formation in 6-OHDA-treated rats were both decreased by L-DOPA administration (Gołembiowska and Dziubina 2012).

In the present study, CSC enhanced L-DOPA-induced increase in extracellular DA and markedly augmented hydroxyl radical production, while ZM 241385 was without effect on either DA or hydroxyl radical level. It seems that CSC by decreasing the MAO-catalyzed DA metabolic rate under conditions of augmented DA synthesis by exogenous L-DOPA raises cytosolic DA pool, and induces its autoxidation and hydroxyl radical overproduction. The increase in hydroxyl radical formation by combination of CSC and L-DOPA was not observed in normal rats (Gołembiowska et al. 2009). Thus, functional storage mechanism is an important factor in proper DA utilization and does not allow for the formation of DA-oxidative products even though neurons are overloaded with exogenous L-DOPA.

The findings of our study indicate that the inhibition of glutamate neurotransmission by A_2A_ receptor antagonists showed also in other studies (reviewed by Popoli et al. 2004) might account for relief of motor symptoms and neuroprotection observed in the models of late-stage PD (reviewed by Chen et al. 2007; Gołembiowska and Dziubina 2012). However, methylxanthine derivatives, such as CSC, bearing properties of MAO-B inhibition may cause a risk of oxidative stress resulting from dysfunctional VMAT2 and DA storage mechanism in an early PD.
